# Concentration Quenching of Fluorescence Decay Kinetics of Molecular Systems

**DOI:** 10.1021/acs.jpcb.3c08254

**Published:** 2024-05-14

**Authors:** Sandra Barysaitė, Jevgenij Chmeliov, Leonas Valkunas, Andrius Gelzinis

**Affiliations:** † Institute of Chemical Physics, Faculty of Physics, Vilnius University, Saulėtekio 9-III, 10222 Vilnius, Lithuania; ‡ Department of Molecular Compound Physics, Center for Physical Sciences and Technology, Saulėtekio 3, 10257 Vilnius, Lithuania

## Abstract

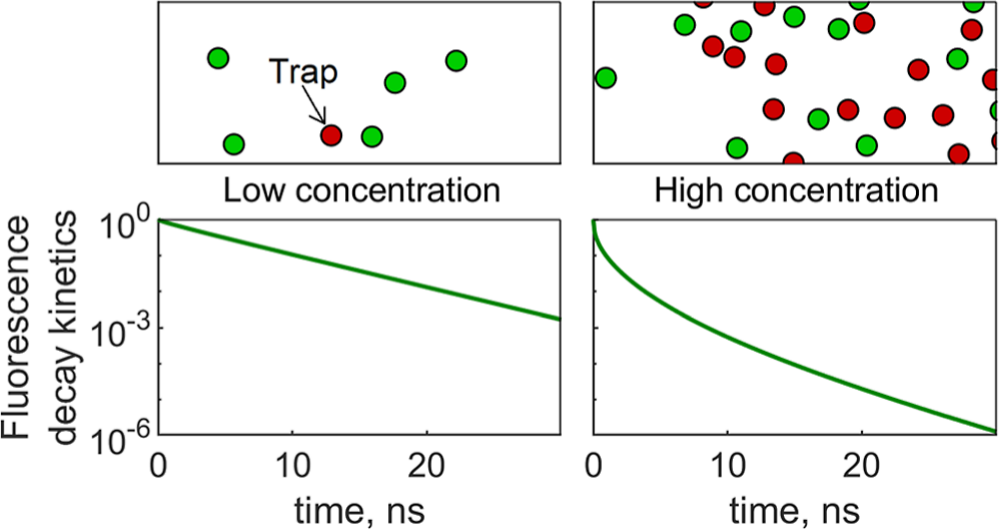

Fluorescence concentration quenching occurs when increasing molecular concentration of fluorophores results in a decreasing fluorescence quantum yield. Even though this phenomenon has been studied for decades, its mechanisms and signatures are not yet fully understood. The complexity of the problem arises due to energy migration and trapping in huge networks of molecules. Most of the available theoretical work focuses on integral quantities like fluorescence quantum yield and mean excitation lifetime. In this work, we present a numerical study of the fluorescence decay kinetics of three-dimensional and two-dimensional molecular systems. We investigate the differences arising from the variations in models of trap formations. We also analyze the influence of the molecular orientations to the fluorescence decay kinetics. We compare our results to the well-known analytical models and discuss their ranges of validity. Our findings suggest that the analytical models can provide inspiration for different ways of approximating the fluorescence kinetics, yet more detailed analysis of the experimental data should be done by comparison with numerical simulations.

## 1 Introduction

Fluorescence concentration quenching is a phenomenon when the fluorescence quantum yield decreases upon increasing molecular concentration. It has been investigated since the middle of the previous century^1−7^ and still remains an active research topic.^8−11^ Concentration quenching has been observed in different chlorophyll systems.^2,12,13^ Rather remarkably, no concentration quenching is observed in *in vivo* photosynthetic systems, even though *in vitro* systems of similar concentrations exhibit severe fluorescence quenching.^14^ Since concentration quenching results in energy loss and lower fluorescence yield, for practical applications, it has to be controlled and limited.^15−17^ This requires a detailed knowledge and understanding of many different facets of this phenomenon. To this day, however, concentration quenching is not yet fully understood.

The complexity of concentration quenching originates in its multilayered nature. For most systems, the global picture is deceptively simple—excitation migrates until a trap is reached. The physical nature of the trap is nonetheless not yet agreed upon. A widely popular statistical pair model assumes that two molecules, closer to each other than a certain critical distance, form a trap.^2^ Formally, as remarked in ref (5), such model does not contain any physical picture since the excitation is just assumed to be lost in the statistical pair. Most prevalent physical explanation of the trapping nature suggests that closely situated molecules, especially the planar ones, tend to form H-type dimers.^18^ Thus, excitation, upon reaching such a dimer, quickly relaxes to the very weakly fluorescent lower excited state. Recently, however, charge transfer was proposed to be responsible for the chlorophyll fluorescence concentration quenching.^11^ On the other hand, trapping can also be assumed to occur due to the acceptor molecules,^19^ which could in principle be of the same species as the fluorescent donors, just affected by the environmental influence. Regardless of the physical nature of the traps, additional source of complexity is due to energy transfer since there are no analytical solutions for energy transfer and trapping in systems of hundreds or thousands of randomly distributed molecules, even assuming the limit of infinitely deep traps.

The theoretical analysis of energy migration and trapping in molecular systems underpins the efforts to understand the concentration quenching phenomenon. Analytical work usually requires a set of approximations. Perhaps the most well-known limit is the case of a single donor molecule surrounded by an infinite number of infinitely deep acceptors.^19,20^ Expressions for two-dimensional (2D), three-dimensional (3D), and even fractal-dimensional systems can be obtained.^4^ Accounting for the energy transfer between the donor molecules is also possible, but the resulting expressions can be rather cumbersome and still require numerical evaluations.^21−24^ All this highlights the importance of fully numerical investigations, which should explicitly account for excitation migration and trapping in specific molecular configurations via kinetic equations or Monte Carlo simulation-based methods. Unfortunately, such works are relatively rare.^18,25,26^

Most theoretical studies focus on the integral quantities, like quantum yield or mean fluorescence lifetime.^2,18,22,25^ The time dependence of the kinetics themselves is analyzed much more rarely. Even though it is simpler to focus on the integral quantities, and the experimental literature usually does so, the information content in these integral quantities is much smaller than in the kinetics. Indeed, as remarked in ref (27), fitting only the mean fluorescence lifetime often does not lead to an unambiguous determination of the model parameter values.

Therefore, here we theoretically analyze the fluorescence decay kinetics in 2D and 3D molecular systems. The kinetics are obtained via numerical solution of the Master equation. We consider both random traps, resulting from the presence of some acceptors in the system, and statistical traps, resulting from the closely situated donor molecules. In addition, we investigate the influence of the molecular orientations, which are often neglected in the analytical work. We compare our results with the simplest analytical model—a single donor surrounded by infinitely deep acceptors—and analyze the deviations from this limit due to energy transfer between the donor molecules.

## 2 Theoretical Methods

In this work, we seek to describe the fluorescence decay kinetics in molecular assemblies. To account for concentration quenching, traps will be included in the model. Assuming that all the fluorescing molecules are of the same species, their transition dipole moment has the same magnitude; thus, the resulting fluorescence signal is proportional to the total excited-state population of the fluorescing species. Therefore, we will focus on the time dependence of this quantity. To this end, we will first provide a theoretical description of the population transfer and decay, which is similar to the one employed in ref (18). To facilitate the analysis of the numerical results, we will then briefly review the known analytical expressions for a simple model of a single-donor molecule surrounded by energetically infinitely deep acceptors. Finally, we will present the details of our numerical model.

### 2.1 Population Decay Kinetics

According to the Förster resonance energy transfer theory, an excited light-sensitive molecule can transfer its excitation energy to another light-sensitive molecule, thus relaxing to the ground state while the other molecule becomes excited.^28,29^ The time dependence of the excitation probability *P*_*i*_(*t*) for each molecule in a system of *N* molecules is obtained by solving the Master equation
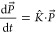
1where  is a vector containing excitation probabilities *P*_*i*_(*t*) of every molecule in the system and  is a matrix of energy-transfer rates between the molecules and relaxation rates to the ground state.

In the Förster limit, the excitation energy transfer rate is proportional to the squared coupling between the molecules. When molecules *i* and *j* are far apart from each other, the coupling between their excited states can be calculated using the dipole–dipole approximation^29,30^
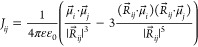
2where ε is the relative permittivity of the medium, ε_0_ is the vacuum permittivity,  is the transition dipole moment of the *i*th molecule, and  is a vector connecting molecules *i* and *j*. However, for simplicity, we use this approximation even for molecules that are close to each other. The magnitude of  is equal for identical molecules , and its direction can be denoted by a unit vector , thus . The magnitude of  is equal to the distance between molecules *i* and *j*, and its direction can also be denoted by a unit vector : . Then, eq 2 can be written simply as
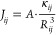
3where *A* = μ^2^/(4πεε_0_) is a constant and κ_*ij*_ is an orientation parameter, expressed as
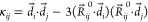
4

Thus, the nondiagonal element of  that describes *i* ← *j* transfer can be written as
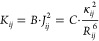
5where *B* and *C* = *A*^2^·*B* are constants. Note that the Förster theory should break down for small intermolecular distances, when excitonic effects or exchange interactions begin to play a larger role. Other works have assumed that for distances smaller than some cutoff, the transfer rate depends on distance differently^18^ or is a constant altogether.^10^ Nevertheless, in order not to overcomplicate our model, we will use the Förster rates for all intermolecular distances.

Excitation can return to the ground state via fluorescence and nonradiative relaxation; thus, the total excitation lifetime of an isolated molecule can be written as
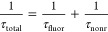
6Moreover, the fluorescence quantum yield (QY) relates the fluorescence lifetime to the total lifetime
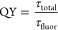
7

The distance, at which the transfer rate *K*_*ij*_ is equal to the fluorescence rate τ_fluor_^–1^, is usually denoted as the Förster radius *R*_F_,^30^ so the constant *C* in eq 5 is expressed as
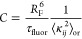
8where  is the squared orientation parameter, averaged over every possible molecular orientation. Using eqs 8 and 5, the nondiagonal elements of  can be written as
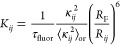
9

Note that the orientationally averaged  in the rate expression does not imply that we assume fast orientational fluctuations. Instead, it arises due to the fact that formally the Förster radius should be different for every orientation between the donor and acceptor transition dipoles; thus, we define it with the average orientational parameter. As an approximation, the molecular orientations could be ignored. In such a case, eq 9 can be written as
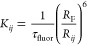
10

Meanwhile, the diagonal elements of  represent the population loss due to the transfer to other molecules and decay to the ground state
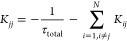
11

To account for concentration quenching, traps were included in the system. We assume the limit of infinitely deep traps. This reduces the number of parameters in the model. In this work, two different methods for trap formation were used. In the random trap model, only the percentage of traps is specified, and randomly chosen molecules become traps. This can be realized when due to the interactions with the surroundings, the properties of a molecule might change (e.g., due to protonation^31^). The statistical trap model assumes that traps are closely situated molecules, with the intermolecular distance less than some threshold value *R*_trap_.^2,5,18^ This limit is often called as the statistical *pair* model, but we want to highlight that in our formulation, not only dimers but also larger aggregates might become traps, which is relevant for larger molecular concentrations. The diagonal elements of  that represent traps and those nondiagonal elements that represent an excitation leaving a trap were set to 0. In both models, energy transfer to the trap molecules is for simplicity described in the same way as energy transfer between the donor molecules.

### 2.2 Analytical Models

To provide a foundation for an easier interpretation of the numerical results, it is worthwhile to consider the simplified cases for which analytical results are available. It is possible to derive an analytical expression for population decay kinetics in a 3D system, which consists of one donor surrounded by infinitely deep acceptors.^19,20^ At the initial moment of time *t* = 0, the donor is excited; thus, the excitation probability is *P*(0) = 1. In the derivation, the molecular orientations are neglected. For a 3D system, the resulting expression for *P*(*t*) is
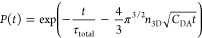
12while for a 2D system
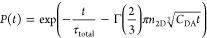
13is obtained.^19,20^ Here, *n*_3D_ (*n*_2D_) is the concentration of the acceptors in the 3D (2D) system and Γ(*x*) denotes the Gamma function. The transfer microparameter *C*_DA_ relates the transfer rate *w*(*r*) from the donor to the acceptor and the distance *r* between them as *w*(*r*) = *C*_DA_/*r*^6^ (cf. with eq 10).

### 2.3 Our Model

Here, we describe the details of our model, which was used to simulate the fluorescence decay kinetics in 2D and 3D molecular systems.

**Figure 1:**
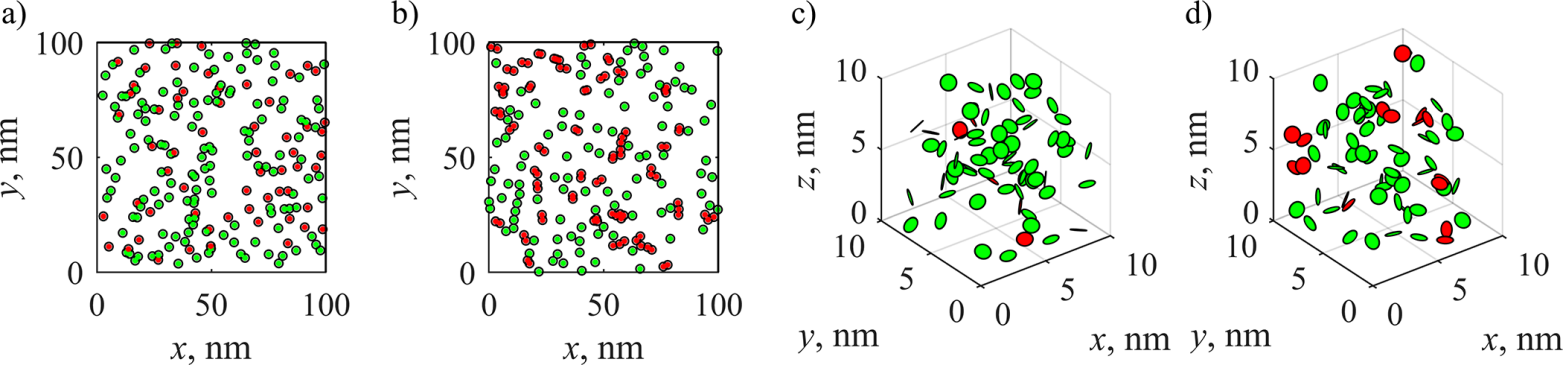
Examples of distribution of molecules, where green and red circles represent fluorescent molecules and traps, respectively. (a) Random trap 2D model, where *n* = 0.02 nm^–2^ and traps make up 30% of the system; (b) statistical pair trap 2D model, where *n* = 0.02 nm^–2^ and *R*_trap_ = 3 nm; (c) random trap 3D model, where *n* = 0.07 nm^–3^ and traps make up 10% of the system; and (d) statistical pair trap 3D model, where *n* = 0.07 nm^–3^ and *R*_trap_ = 1 nm. In the case of the 2D model, molecules are shown to be larger than 1 nm for clarity.

In the 2D model, *N* molecules, each 1 nm in diameter, were scattered randomly across a 100 nm × 100 nm area using a uniform distribution. The size of the molecules was taken to be similar to the porphyrin ring system of chlorophylls. Different concentrations *n* were obtained by changing *N*; the chosen values were based on the experimental data from ref (13), where the fluorescence lifetimes of chlorophylls in monolayers were measured. Figure 1a,b demonstrates the examples of such a system for random and statistical formation models. The values of the fluorescence quantum yield and the total excitation lifetime for calculating τ_fluor_ from eq 7 were set to QY = 0.33 and τ_total_ = 5 ns,^32^ and the value of the Förster radius was *R*_F_ = 5 nm. The value of *R*_trap_ in the 2D model was 3 nm. The direction of the dipole moment  for each molecule was determined by picking an angle from an interval [0; 2π) using a uniform distribution, and the value of  for a 2D space is 5/4. Calculated population decay kinetics were averaged over the random positions of the molecules.

The same principle was used in the 3D model, where molecules were scattered uniformly in a 20 nm × 20 nm × 20 nm box. Figure 1c shows an example of such molecular distribution using a random trap formation model and Figure 1d using the statistical trap model. The values of QY, τ_total_, and *R*_F_ were the same as in the 2D case; however, the value of  for a 3D space is 2/3. The values of concentration *n* were chosen based on the experimental data from ref (3), where chlorophyll fluorescence lifetimes in lipid liposomes were measured. In the 3D case, the value of *R*_trap_ was chosen to be 1 nm. Note that significantly different values for *R*_trap_ for 3D and 2D models were chosen to explore different points in the parameter space and not to represent specific situations.

Having a complete transfer rate matrix ,  is then calculated by solving eq 1. At the initial moment of time *t* = 0, probability is equally distributed over the fluorescent molecules. This corresponds to excitation of a molecular system by an infinitely short laser pulse. Elements of  that correspond to the fluorescent molecules are summed up to obtain the total probability decay kinetics *P*_sum_(*t*). In every case, the results were averaged over 100 different distributions of molecules.

## 3 Results

In this work, we are interested in the behavior of the excited-state population decay kinetics that should mimic the experimentally observable fluorescence signal. We will mostly focus on two points. First, how slight changes in the model formulation (inclusion of molecular orientations, random or statistical traps, etc.) influence the kinetics. Second, how close the obtained kinetics are to the available analytical models.

### 3.1 Kinetics in 2D Systems

Let us first consider the simplest model—random traps with molecular orientations not included in the transfer rates.

**Figure 2:**
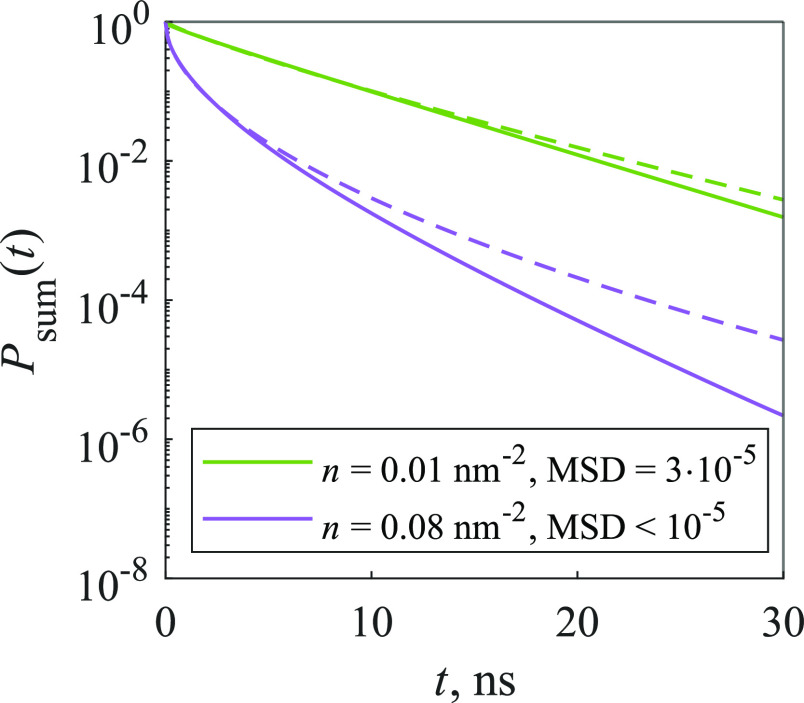
Calculated kinetics of the 2D model (solid lines), approximated using eq 14 (dashed lines). Traps make up 30% of the system.

We have calculated the population decay kinetics for various parameter values, and they were nonexponential. Thus, the stretched exponential function was used to fit the calculated kinetics
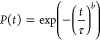
14where τ and *b* are parameters, whose values were changed during the optimization process. This is a simple description of the nonexponential behavior, and it was applied recently to the FL signal of thin films of zinc-phthalocyanine.^10^Figure 2 illustrates that the stretched exponential function can fit the calculated kinetics sufficiently well, as the mean squared deviations (MSDs) between the function and the kinetics are fairly small (∼10^–5^). While this demonstrates the complexity of the calculated kinetics and serves as an example of the suitability of the stretched exponential description, in the general case other descriptions could nevertheless be more easily interpretable.

Thus, as an alternative description of the decay curves, eq 14 was modified to explicitly include the finite lifetime of an isolated molecule
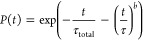
15

This type of description is inspired by the analytical results summarized in Section 2.2.

**Figure 3:**
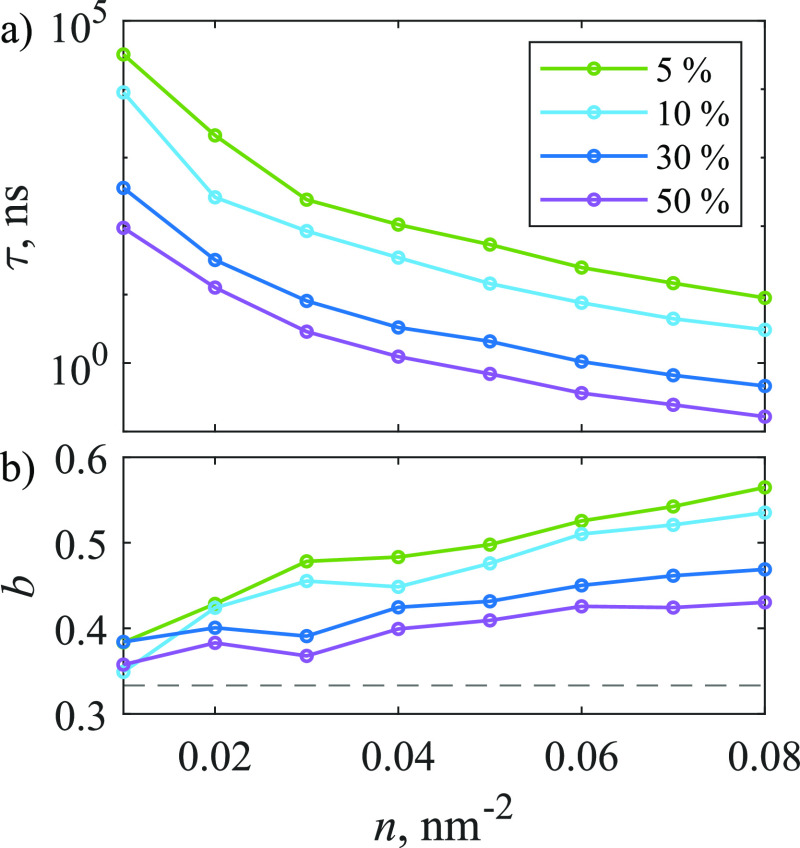
Dependence of eq 15 parameters (a) τ and (b) *b* on the molecular concentration and the percentage of traps in the 2D model. Dashed line at *b* = 1/3 corresponds to the value expected from eq 13. Note that the *y* axis in part (a) is logarithmical.

The values of MSD for different molecular and trap concentrations, obtained using eq 15, were small (∼10^–6^). The dependence of parameters τ and *b* on concentration is shown in Figure 3: the value of τ decreases for larger concentrations and the amount of traps, while the value of parameter *b* increases with increasing concentration values, though this increase is smaller for larger amount of traps.

The obtained values of parameter *b* are fairly close to 1/3, as shown in Figure 3b, especially for higher trap percentages; therefore, we fixed *b* to this value, thus making the fitting function equivalent to eq 13
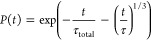
16

In this case, the values of MSD for higher concentrations of molecules and traps are larger than they were using eq 15 (10^–4^–10^–5^); however, when these concentrations are low, the model represents the ideal system that is described in ref (4), and the values of MSD reach 10^–6^.

**Figure 4:**
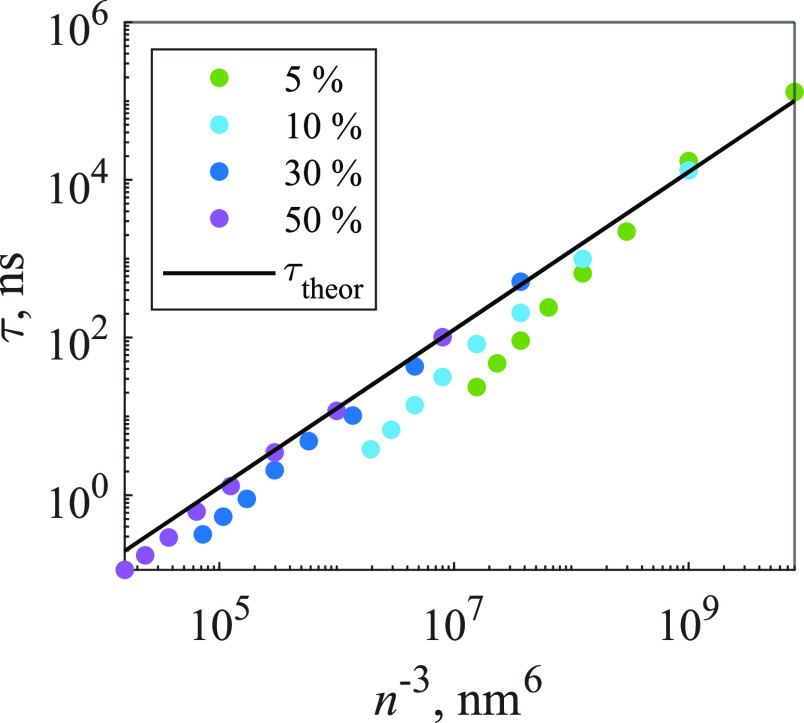
Dependence of eq 16 parameter τ values on the molecular concentration and the percentage of traps (indicated in the legend) in the 2D model. Dots represent τ and solid line represents τ_theor_.

The dependence of eq 16 parameter τ on concentration is shown in Figure 4. Since it is interesting to compare numerical results with analytical predictions, we derive the theoretical dependence of the parameter τ_theor_ on the acceptor concentration *n*_2D_ by equating eqs 13 and 16
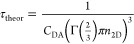
17with *C*_DA_ = *R*_F_^6^/τ_fluor_, as discussed above. We assumed that in this case, traps represent acceptors; thus, the molecular concentrations that are shown in Figure 4 were accordingly multiplied by 0.05, 0.1, 0.3, and 0.5 in order to obtain the concentrations of traps, which were used to calculate different values of τ_theor_; results are compared with τ in Figure 4. The agreement of the numerical values with the theoretical curve is better for larger trap percentages and smaller molecular concentrations. Indeed, calculated curves that correspond to the same concentrations of traps were very close in the low concentration range. The deviations get larger for smaller trap percentages because then the model does not satisfy the assumptions behind eq 13.

**Figure 5:**
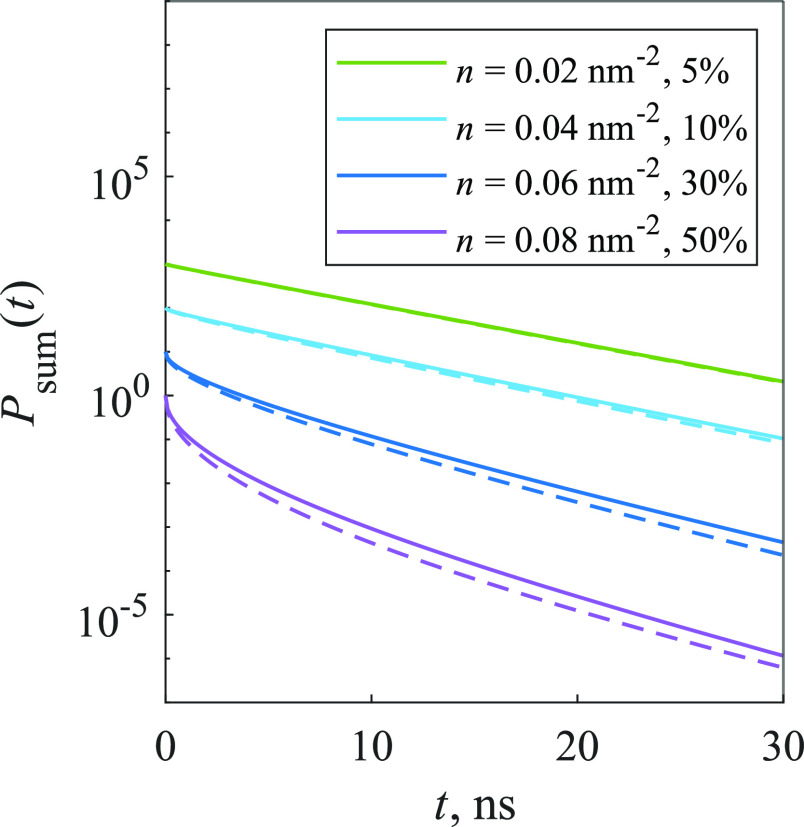
Comparison of kinetics of the 2D model when molecular orientations are either included (solid line) or not included (dashed line). Percentages represent the amount of traps in the system. Curves representing different parameter values were multiplied by powers of 10 for clarity.

Next, we investigated the influence of the molecular orientations to the population decay kinetics. Thus, eq 9 was used to calculate the nondiagonal elements of . Figure 5 shows a comparison between the kinetics with and without accounting for molecular orientations. Even though it is clear that quenching is faster in the latter case, there are no significant differences even for higher concentrations.

**Figure 6:**
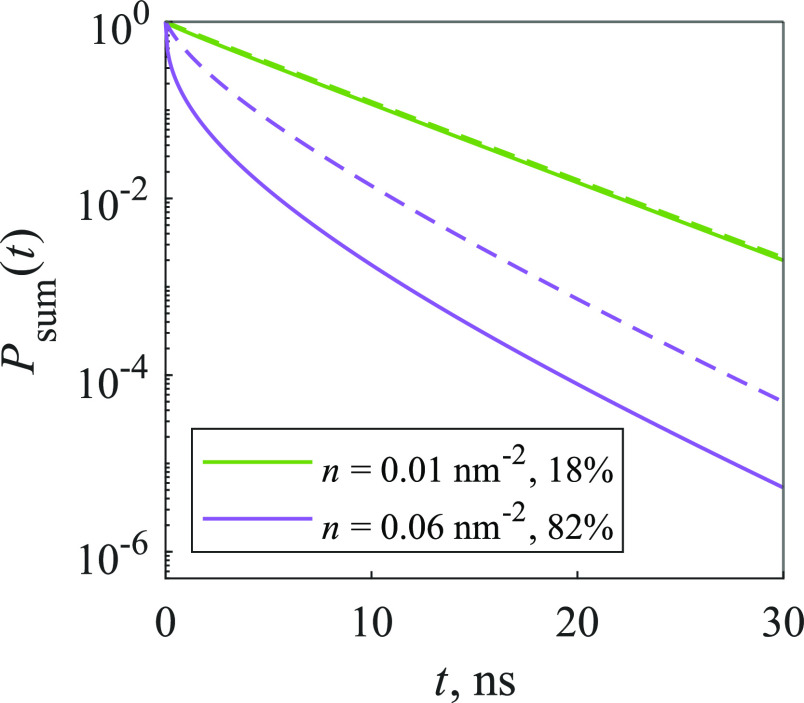
Comparison of kinetics of random (solid line) and statistical (dashed line) trap 2D models. Percentages represent the amount of traps in the system.

Finally, we investigated the statistical trap model, where traps are assumed to form in closely situated pairs of molecules (or larger aggregates). In order to compare the kinetics of both trap models, at first we calculated the kinetics of the statistical trap model, then we evaluated the percentage of traps in the system, and this percentage was used to calculate the kinetics of the random trap model. Figure 6 compares these two models: when the concentration is low, the kinetics are almost identical, but as the concentration gets higher, differences begin to appear—quenching is slower in the statistical trap model. This can be explained by the distribution of traps in the system: when traps are taken to form in the statistical pairs or larger aggregates, they accumulate in certain areas; thus, excitation has to travel further to reach a trap compared to a random trap model, where traps are distributed uniformly.

### 3.2 Kinetics in 3D Systems

Similarly to the 2D model, at first the simple 3D model with random traps was considered. Equations 11 and 10 were used to calculate the elements of  that represent the fluorescent molecules.

**Figure 7:**
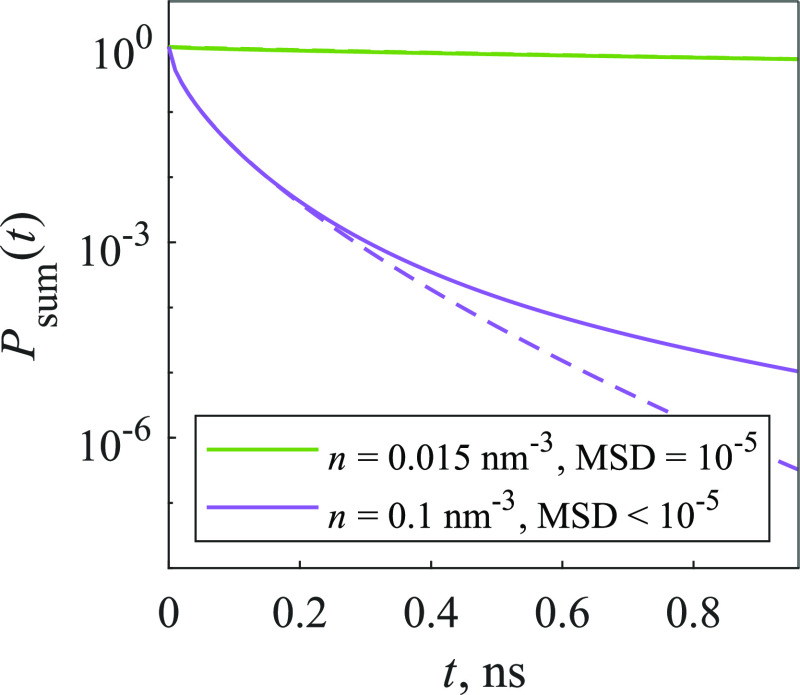
Kinetics of the 3D model (solid lines), approximated using eq 14 (dashed lines). Traps make up 5% of the system for *n* = 0.015 nm^–3^ concentration and 30% for *n* = 0.1 nm^–3^ concentration.

First, kinetics of such model were fitted using eq 14, and results are shown in Figure 7. The fitting quality is quite good using the stretched exponential, as the values of MSD using this function are fairly low (MSD ≤ 10^–5^).

**Figure 8:**
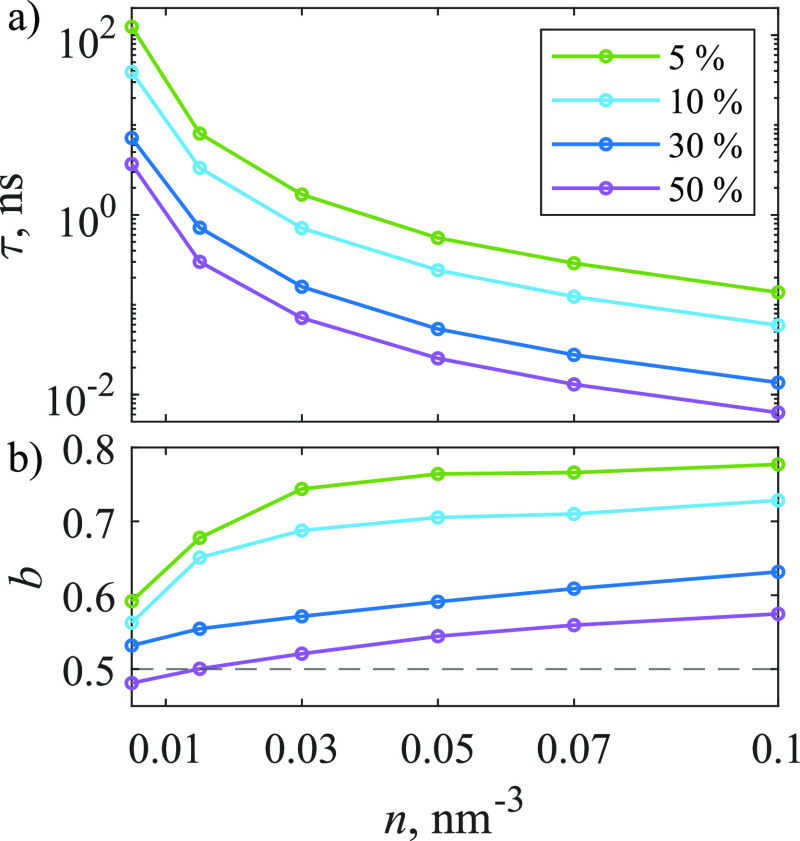
Dependence of eq 15 parameters (a) τ and (b) *b* on molecular concentration and the percentage of traps in the 3D model. Dashed line at *b* = 1/2 corresponds to the value expected from eq 12.

Equation 15 helped to achieve better approximation accuracy for 2D systems; therefore, it was also used to fit the kinetics of 3D systems. In this case, the values of MSD are close to those obtained using eq 14—also equal to or less than 10^–5^. The dependence of parameters τ and *b* on concentration shown in Figure 8 is similar to Figure 3, as with increasing concentration τ decreases very rapidly and *b* slowly increases.

The obtained values of parameter *b* are relatively close to 1/2, as shown in Figure 8b; therefore, just like in the case of a 2D model, we fitted the kinetics using a function that is equivalent to eq 12 which describes the kinetics of an ideal 3D system with one donor surrounded by *multiple* acceptors
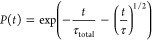
18

When the concentration of traps is high, the calculated kinetics and the fitted curve are very close (MSD ≤ 10^–5^) for each molecular concentration value: this case represents the ideal 3D model, described in refs (4 and 19). For lower concentration of traps, the values of MSD are larger: 10^–5^–10^–3^.

**Figure 9:**
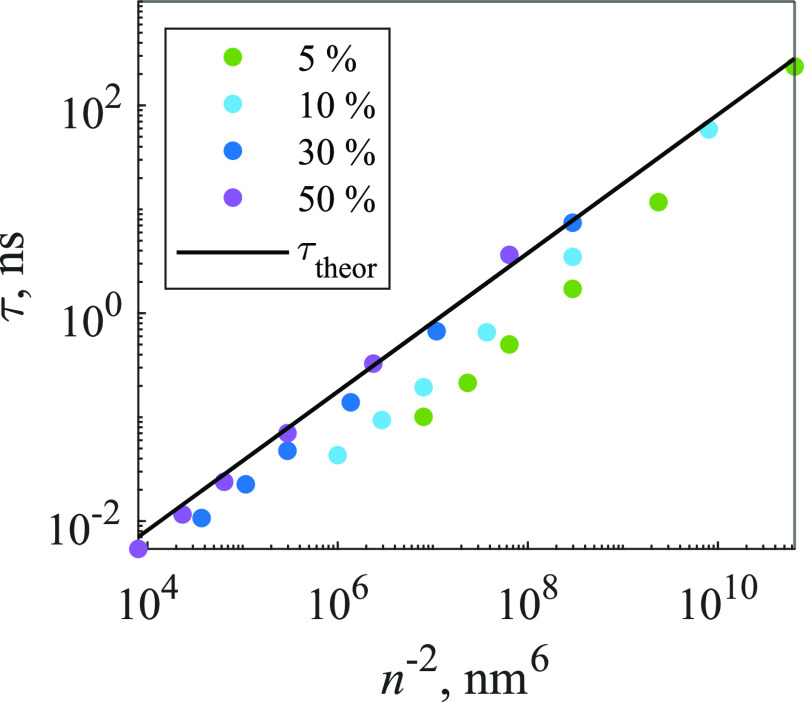
Dependence of eq 18 parameter τ on molecular concentration and the percentage of traps (indicated in the legend) in the 3D model. Dots represent τ and solid line represents τ_theor_.

The dependence of τ on concentration is shown in Figure 9. Again, by equating eqs 12 and 18, we derive the theoretical dependence of parameter τ_theor_ on the acceptor concentration *n*_3D_
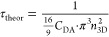
19with *C*_DA_ = *R*_F_^6^/τ_fluor_, as in the 2D case. Trap concentrations for the calculation of τ_theor_ were obtained in the same way as in the 2D system. Comparison between τ and τ_theor_ values is shown in Figure 9. As expected, the differences between both curves are less significant for higher trap percentages and lower molecular concentrations. In the case of smaller trap percentages, the deviation from the theoretical prediction increases considerably.

**Figure 10:**
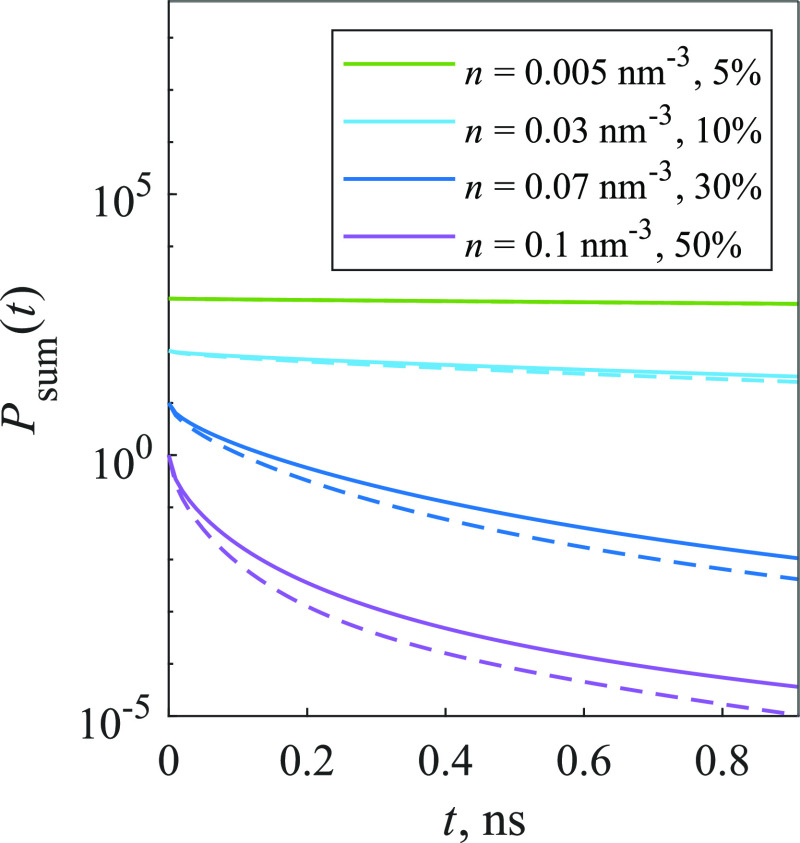
Comparison of kinetics of the 3D model when molecular orientations are included (solid line) and are not included (dashed line). Percentages represent the amount of traps in the system. Curves representing different parameter values were multiplied by powers of 10 for clarity.

The comparison between kinetics with and without accounting for molecular orientations was done similarly to the 2D case, with eq 9 being used to calculate the nondiagonal elements of , and it is demonstrated in Figure 10. The result is also similar: quenching is slightly slower when the molecular orientations are included in the energy-transfer rates.

**Figure 11:**
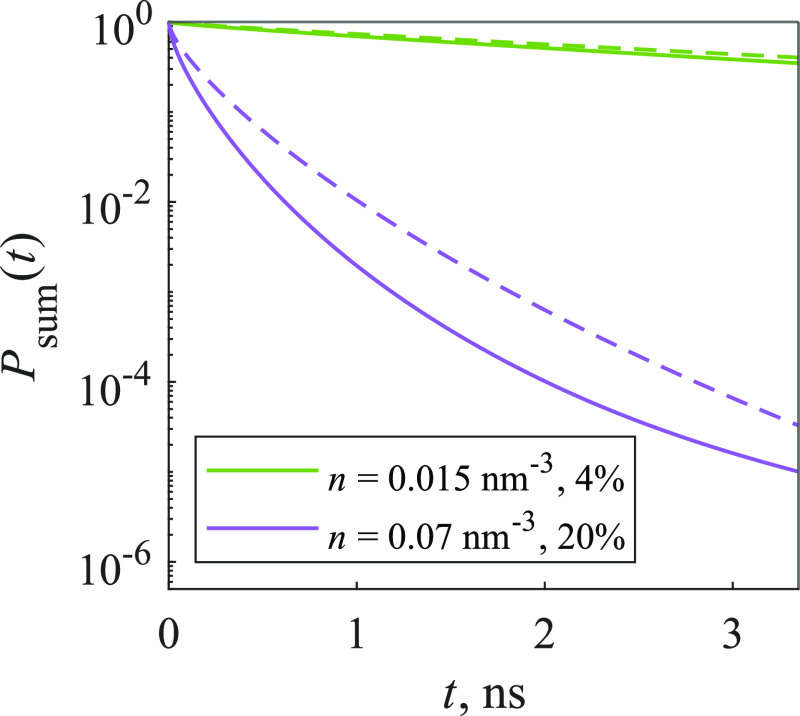
Comparison of kinetics of random (solid line) and statistical pair (dashed line) trap 3D models. Percentages represent the amount of traps in the system.

Last, we compared the random trap 3D model with the statistical trap model, where the percentage of traps was calculated as described in the 2D case. Results are shown in Figure 11. As expected, quenching is slower in the statistical trap model, which could be explained as in the 2D model—the distribution of traps causes excitation to travel further to reach a trap in the statistical trap model and less in the random trap model.

### 3.3 Application

As an application of the present approach, here we consider fluorescence concentration quenching in solutions of disulfonated aluminum phthalocyanine (AlPcS_2_).^33^ To model the fluorescence decay kinetics, we utilize the 3D model with statistical traps, as evidence for dimer formation is clear in the experimental data.^33^ To simulate the experimental conditions, the diameter of molecules was taken to be 1.5 nm, the quantum yield was set to QY = 0.4, and the value of *R*_trap_ was chosen to be 4.5 nm. The total lifetime τ_total_ was set to 5.0 ns. To compare our calculations with the experimental data, we considered molecular concentrations of 0.00031, 0.00062, 0.00124, 0.00186, 0.00248, 0.00372, 0.00496, 0.0062, and 0.0124 nm^–3^ (corresponding to 0.5, 1, 2, 3, 4, 6, 8, 10, and 20 mM, respectively). The molecules were scattered uniformly in a 50 nm × 50 nm × 50 nm box. The calculated fluorescence decay kinetics were averaged over 100 different random distributions of molecules and then convolved with the instrument response function that is taken to be of Gaussian form with the full width at half-maximum of 0.1 ns and centered at *t* = 3.5 ns.

**Figure 12:**
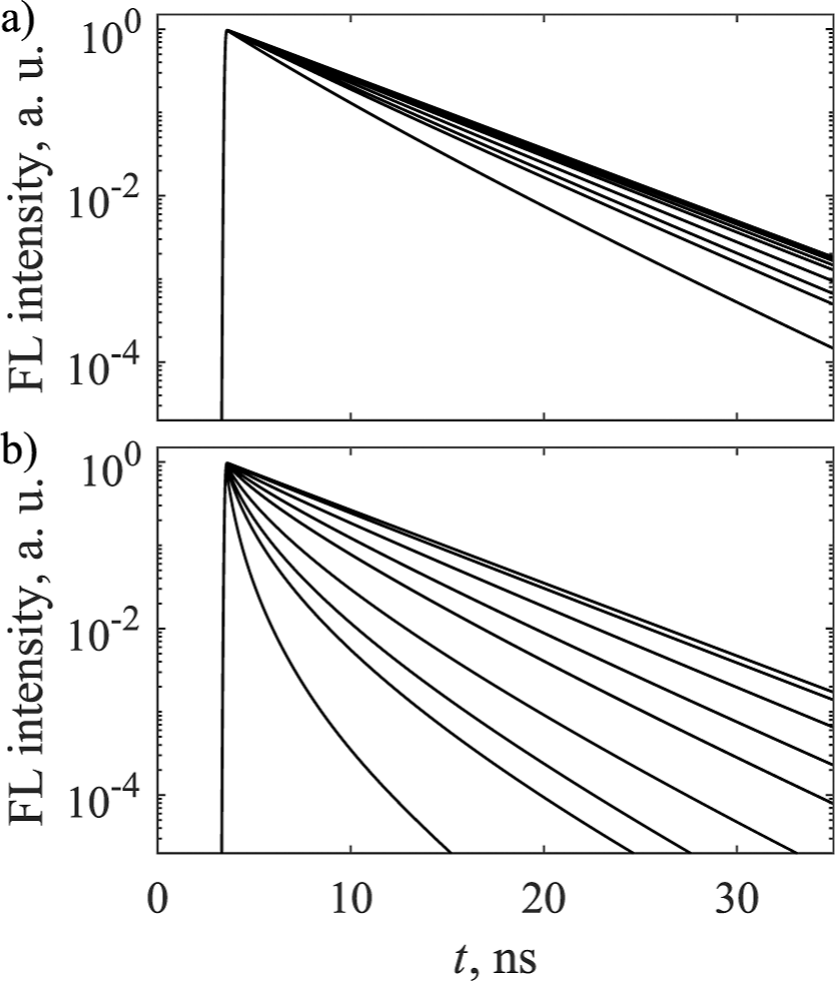
Kinetics of the 3D model with statistical traps, when the value of the Förster radius is (a) *R*_F_ = 4 nm and (b) *R*_F_ = 7 nm. Faster quenching corresponds to higher concentrations, which are as follows: 0.00031, 0.00062, 0.00124, 0.00186, 0.00248, 0.00372, 0.00496, 0.0062, and 0.0124 nm^–3^.

In Figure 12, we present calculations corresponding to *R*_F_ = 4 nm and *R*_F_ = 7 nm. They are in quite good agreement with the experimental fluorescence decays of AlPcS_2_in PBS at pH = 7.4 and pH = 11.5 as given in Figures 5 and 6 of ref (33), with the larger Förster radius corresponding to the higher pH. From the absorption spectra of the same solutions given in ref (33), it appears that in the case of lower pH, more dimers are formed in the solution. The smaller value of *R*_F_ provides an indication that it is harder for the excitation to reach the traps. This might be related to the slower transfer between monomers and dimers than between monomers only, as was also estimated in ref (33).

Clearly, the above consideration should be viewed as an effective model of the problem. Nonetheless, it already provides insights into the concentration quenching of fluorescence in AlPcS_2_ solutions.

## 4 Discussion

In this paper, we numerically investigated concentration quenching in 2D and 3D molecular systems. Even though we kept our models as simple as possible, the signatures of complexity of this phenomenon are already apparent. We will now discuss these issues in turn.

Let us begin by considering the functional form of the calculated kinetics and different ways to parametrize them. Formally, a solution of a system of kinetic equations could be expressed as a sum of decaying exponential functions, with the number of terms equal to the number of molecules in the system. Hundreds of parameters would then be required. Of course, the information content in the decay curves is much smaller, as they could be approximated by a simpler functional form. In the presence of energy transfer, the kinetics show deviations from single exponential behavior. A simplest way to characterize these deviations is to assume a stretched exponential decay law, which increases the functional freedom at the cost of an additional model parameter.^34^ We tried to approximate our obtained population kinetics with a stretched exponential function for both 2D and 3D systems and found that it provides a good agreement with the calculations. It is therefore a good choice for the initial data analysis. Nevertheless, the approximated decay curves exhibit some differences from the calculated ones, especially at longer times. This could become important, for example, when fitting experimental fluorescence data with high signal-to-noise ratio. In the case of comparisons with the experiment, it is thus best to use the actual calculated curves rather than their approximations. We also note that we recently investigated the fluorescence decay kinetics in a 2D lattice system, and in that case, a sum of two stretched exponential functions was needed to adequately describe the data.^35^

In our view, inspiration for different functional forms could be drawn from the available theoretical models. We had some success in using a form where the donor lifetime was included explicitly and the stretched exponential part remains, see eq 15. Approximation accuracy equal or better than using the simple stretched exponential was achieved. The donor lifetime should be available experimentally from the measurements corresponding to low concentrations, where no concentration quenching occurs. We therefore suggest that this type of decay function could be useful for description of experimental data as well. It is important to note that by fixing the donor lifetime, eq 15 has only two free parameters, the same as in the simple stretched exponential decay. Thus, overfitting can be avoided. Additional freedom in describing the decay could be achieved by changing the τ_total_ in eq 15 to a free parameter since it was shown previously that accounting for excitation diffusion in an approximate way could lead to such changes.^23,36^ Of course, the best approximation could be achieved by postulating a sum of simple or stretched exponential functions, but this would also result in a complete loss of interpretability of the obtained parameter values.

We now turn to the applicability of the analytical models. The model of a single donor surrounded by infinitely deep acceptors has been widely employed to analyze and interpret the experimental data on fluorescence quenching.^37,38^ Most of the considered systems, however, cannot be taken to include only a single donor. Thus, in the present work, we investigated deviations from the simplest model in the presence of energy transfer between the donor molecules. Our results have shown that in the limit of very small molecular concentration, eqs 12 and 13 provide an adequate description of the population decay kinetics in 3D and 2D systems, respectively. At larger molecular concentrations, deviations between the analytical results and simulations become apparent. Moreover, these deviations are much larger for smaller relative trap concentrations. Since at a larger trap concentration excitation reaches the traps faster, with less hoppings between the donor molecules, the effects of energy transfer between the donor molecules are much less important, and the system is closer to a model system with a single donor. Nevertheless, these simple models have a limited range of validity; thus, in-depth theoretical simulations should explicitly include all the population transfer possibilities, either via kinetic equations, as is the case in this work, or using Monte Carlo-type approaches, as in ref (18). Interestingly, it was shown previously that neglecting the possibilities of a long-range energy transfer, which is a numerically attractive approximation, can lead to noticeable deviations from the full calculations.^25^

It is also worthwhile to mention that for the ideal system of a single donor, the law of population decay remains the same regardless of molecular concentration. Our simulations clearly show that excitation migration between the donor molecules makes the decay law concentration dependent, as the power of time in the exponent changes (see Figures 3 and 8). This is a demonstration of complexity resulting from the large number of degrees of freedom in the system. Moreover, this highlights the importance of obtaining the experimental data over a very broad concentration range, even though for some systems (e.g., chlorophylls in solutions), it is very difficult to reach the required molecular concentrations and still obtain undistorted fluorescence signal due to the inner filter effects.^39^

Let us now discuss the effects of molecular orientations in the model. We analyzed the influence of the molecular orientations for both 2D and 3D systems. For both cases, their effects upon the total population decay kinetics are minor, but inclusion of the orientations in the model results in slower decay (see Figures 5 and 10). This can be explained by the fact that for specific orientations, the Förster transfer rate can be reduced to zero regardless of the distance between the molecules. Thus, even at very large concentrations, there is a nonvanishing probability that some molecules could be effectively isolated, thus slowing down the total population decay. The distribution of the relative Förster transfer rates for a fixed intermolecular distance but different possible orientations was investigated in ref (5). It was shown that this distribution is quite wide and skewed. Our numerical results show that these effects do not contribute to the energy transfer dynamics as much as could be expected, but they are definitely relevant. Overall, we conclude that for purely theoretical simulations or initial assessment of experimental data, the molecular orientations could be neglected, but more detailed models should include them.

We consider the trap model next. Due to the differences in model formulation, it is not straightforward to compare the random and statistical trap models. Formally, neither model is simpler than the other because one parameter in the random trap model (percentage of traps) is replaced by another parameter in the statistical trap model (trap distance). We have made the comparison by first considering the statistical trap model, calculating the mean trap percentage at some fixed molecular concentrations, calculating the population decay kinetics and then doing the calculation with the random trap model with the same trap percentage. Our results show that the population decays slower in the statistical pair model (see Figures 6 and 11). This can be explained by considering that at least two molecules are needed to form a trap in the statistical trap model; thus, at the same formal trap percentage, the traps are much more clustered in the system, and the excitation has to travel further to reach the trap. This can be easily seen in the molecular distributions presented in Figure 1. The choice of the model in simulations thus should reflect the relevant physics of the system under consideration.

Interestingly, our work has revealed no qualitative differences between 2D and 3D molecular systems. In both cases, we clearly see quenching upon increasing molecular concentration, the range of validity for the isolated donor model is similar, effects of molecular orientations are the same, and differences between random and statistical pair trap models are also very similar. Therefore, within our assumptions and approximations, the behavior of the 2D and 3D molecular systems is qualitatively the same. This does not mean, however, that there could not be any differences in principle. Indeed, if we were to assume that quenching in statistical pairs is due to a formation of H-type dimers, like in ref (18), then we could expect larger differences in 2D and 3D systems arising from the available geometric configurations. Still, our results show quantitative difference in the power of time in the exponent of the approximated form of the population kinetics, as illustrated in Figures 3 and 8, with parameter *b* being about 1.5 larger for 3D systems, as expected from the analytical results.

Let us discuss the limitations of our model. Perhaps, the most important point is that we assume that the traps are infinitely deep. In principle, this limit could be reached in two cases—either the internal trapping rate, describing the excitation relaxation from the excited to the ground state, is very fast, or population back-transfer from the trap to neighboring molecules is very slow. We focused on this limit for two reasons. First, this is assumed in the analytical model of a single donor surrounded by acceptors, and we wished to compare our results with this model. Second, relaxing this approximation would require us to introduce two additional parameters in the model, describing the internal trapping rate and population back-transfer rate. If needed to describe actual experiments, however, the model could be easily extended to include these parameters. We note that in our recent investigation of the fluorescence concentration quenching in thin films of zinc-phthalocyanine, we concluded that the back-transfer from the traps has to be accounted for.^10^ Thus, this has to be kept in mind when modeling real experimental data. As demonstrated in Section 3.3, even in this quite simplified form, the present model can already provide insights to the relevant physical situations.

In this work, we have focused on the population kinetics that result from different trap models, rather than the physical basis of the said models. On the one hand, this makes our work compatible with many physical mechanisms. On the other hand, it can give no insight into the physical nature of the traps, provided that they can be described by our considered limits.

The random trap model should represent a case when only one molecular species is present, and due to the interaction with the environment (solvent, protein matrix, etc.), a part of such molecules have their properties changed. The model considered here requires that such trapping molecules should have their absorption/emission maxima shifted, so that they would not get excited by the laser pulse targeting the main absorption band of the system. Also, the model assumes that such molecules should act as infinitely deep energy traps. While these requirements appear strict, they can be realized in actual physical systems. For example, in ref (31), it was shown that zinc-phthalocyanine molecules undergo sequential protonation in acidic ethanol solution, which shifted their emission maxima and reduced the fluorescence quantum yield. As another possibility, the random trap model could be realized if the system under consideration is a binary mixture of molecules, with one species possessing shifted absorption/emission spectra and a shorter excited-state lifetime.

Regarding the statistical trap model, it is mostly related to the formation of the statistical pairs, which are assumed to quench the excitation. Close proximity of neighboring molecules should result in shifts of the excited-state energy levels; thus, such molecules might not be excited by the excitation laser pulse. The actual mechanism of energy trapping in such closely situated molecules is still under debate to this day. Most often, it is assumed that the trapping results from the formation of H-type dimers or higher aggregates, as in ref (18). On the other hand, recent work demonstrates that charge-transfer states might be responsible for excitation quenching,^11^ as was suggested earlier.^40^

## 5 Conclusions

In conclusion, here we numerically investigated the fluorescence concentration quenching in 2D and 3D systems. Our results demonstrated nonexponential decay behaviors that could be approximated using a stretched exponential function. Better accuracy, however, is obtained by utilizing expressions inspired by the analytical results. The model of a single donor surrounded by infinitely deep acceptors can be used to interpret the data for small molecular concentrations and large trap percentages, but its range of validity is limited, and in the general case, fully numerical simulations should be used instead. Influence of the molecular orientations to the energy transfer rates should not be neglected. Both random and statistical trap models can be applied to describe the quenching. For the same trap percentage in the system, the random trap model results in faster quenching. The choice of the trapping model should depend on the physics under consideration.

Our numerical work demonstrated the richness of possible excitation decay behaviors. Future studies, aimed at the elucidation of the physical quenching mechanisms, should thus consider not only integral parameters like fluorescence quantum yield or mean excitation decay time scale but also the time-dependence of the fluorescence decay kinetics, in order to constrain the considered models.
